# Carotid artery dissection and motor vehicle trauma: patient demographics, associated injuries and impact of treatment on cost and length of stay

**DOI:** 10.1186/s12873-016-0088-z

**Published:** 2016-07-08

**Authors:** Jared E. Kray, Viktor Y. Dombrovskiy, Todd R. Vogel

**Affiliations:** Division of Vascular Surgery, University of Missouri, School of Medicine, Columbia, MO USA; Department of Surgery, Rutgers-Robert Wood Johnson Medical School, New Brunswick, NJ USA; Department of Surgery, Division of Vascular Surgery, University of Missouri Hospital & Clinics, One Hospital Drive, Columbia, MO 65212 USA

## Abstract

**Background:**

Blunt carotid arterial injury (BCI) is a rare injury associated with motor vehicle collision (MVC). There are few population based analyses evaluating carotid injury associated with blunt trauma and their associated injuries as well as outcomes.

**Methods:**

The Nationwide Inpatient Sample (NIS) 2003–2010 data was queried to identify patients after MVC who had documented BCI during their hospitalizations utilizing ICD-9-CM codes. Demographics, associated injuries, interventions performed, length of stay, and cost were evaluated.

**Results:**

1,686,867 patients were estimated having sustained MVC; 1,168 BCI were estimated. No patients with BCI had open repair, 4.24 % had a carotid artery stent (CAS), and 95.76 % of patients had no operative intervention. Age groups associated with BCI were: 18–24 (27.8 %), 47–60 (22.3 %), 35–46 (20.6 %), 25–34 (19.1 %), >61 (10.2 %). Associated injuries included long bone fractures (28.5 %), stroke and intracranial hemorrhage (28.5 %), cranial injuries (25.6 %), thoracic injuries (23.6 %), cervical fractures (21.8 %), facial fractures (19.9 %), skull fractures (18.8 %), pelvic fractures (18.5 %), hepatic (13.3 %) and splenic (9.2 %) injuries. Complications included respiratory (44.2 %), bleeding (16.1 %), urinary tract infections (8.9 %), and sepsis (4.9 %). Overall mortality was 14.1 % without differences with regard to intervention (18.5 % vs. 13.9 %; *P* = 0.36). Stroke and intracranial hemorrhage was associated with a 2.7 times greater risk of mortality. Mean length of stay for patients with BCI undergoing stenting compared to no intervention were similar (13.1 days vs. 15.9 days) but had a greater mean cost ($83,030 vs. $63,200, *p* = 0.3).

**Conclusion:**

BCI is a rare injury associated with MVC, most frequently reported in younger patients. Frequently associated injuries were long bone fractures, stroke and intracranial hemorrhage, thoracic injuries, and pelvic fractures which are likely associated with the force/mechanism of injury. The majority of patients were treated without intervention, but when CAS was utilized, it did not impact mortality and trended toward increased costs.

## Background

Blunt carotid arterial injury (BCI) is a rare injury associated with motor vehicle collision (MVC). Biffl et al. recognized that blunt carotid arterial injuries were associated with closed head injuries, facial fractures, and thoracic injuries in patients admitted to their center [1]. Few population based analyses evaluating injuries associated with carotid artery dissection secondary to blunt trauma exist. It is estimated that it occurs in <1 % to 3 % of MVC and dissections are estimated to only account for 2 % of ischemic strokes in total [2]. However, in the younger patients aged less than 45 years, it accounts for disproportionate 20 % of all cause ischemic strokes [3].

The objective of this population level analysis was to describe the reported frequency of BCI, delineate associated injuries, evaluate the interventions performed, and assess the outcomes of all patients across the country with documented blunt carotid arterial injury after MVC by querying the Nationwide Inpatient Sample (NIS) 2003–2010 data.

## Methods

A secondary analysis of the Nationwide Inpatient Sample (2003–2010) was performed. To identify external cause of injury we used the following ICD-9-CM (International Classification of Diseases, Ninth Revision, Clinical Modification) [4] E- codes: motor vehicle crash – driver E811-E816(.0); motor vehicle crash – passenger E811-E816(.1); motorcycle – driver E811-E816(.2); motorcycle – passenger E811-E816(.3); and pedestrian E811-E816(.7). Patients with dissection of the carotid artery were identified with the ICD-9-CM diagnosis code443.21.

With the appropriate ICD-9-CM diagnosis codes (Attachment 1) we also identified the most common injuries seen in blunt trauma patients including: cranial, vertebral column with spinal cord injury, intrathoracic organs (lungs, bronchi and esophagus), small bowel, colon, spleen, kidney and pelvic organs (ureter, bladder and urethra), liver, long bone fractures, pelvic fractures, fractures of the base of the skull, fractures of facial bones, fractures of the cervical vertebrae without spinal cord injury and clavicle fracture.

All patients with BCI were classified into three treatment groups: open surgery (ICD-CM procedure codes 38.02, 38.l2, 38.32, 38.42, 38.62, 38.82), carotid stenting (00.63), or non-operative treatment (all the other). These patients were then analyzed for hospital complications (Attachment 2), hospital mortality, length of stay (LOS), and total hospital cost. Secondary analysis was performed on patients diagnosed with BCI and ICD-9 CM diagnosis codes were then used to classify associated injury types (Tables 1 and 2). The study was approved by the Institutional Review Board at both institutions. Informed patient consent was not needed as the Nationwide Inpatient Sample database is de-identified. As well, none of the authors have any competing interests.

**Table 1:** ICD-9-CM diagnosis codes for most common injuries

Multiple rib fractures	807.09, 807.19, 807.4
Small bowel	863.20, 863.21, 863.29
Colon	863.40-863.46, 863.49
Liver	864.00-864.05, 864.09, 864.10-864.15, 864.19
Intrathoracic organs (lungs, bronchi, esophagus)	861.20-861.22, 862.0, 862.21, 862.22, 862.29
Spleen	865.00-865.04, 865.09
Kidney and pelvic organs	866.00-866.03, 867.0, 867.2
Vertebral column with spinal cord injury	806.00-806.09, 806.10-806.19, 806.20-806.29, 806.30-806.39, 806.4, 806.5, 806.60-806.62, 806.69, 806.70-806.72, 806.79
Cranial	852.02-852.06, 852.12-852.16, 852.22-852.26, 852.32-852.36, 852.42-852.46, 852.52-852.56, 853.02-853.06, 800.22-800.26, 800.72-800.76, 801.22-801.26, 801.72-801.76
Long bones fractures	820.00-820.03, 820.09, 820.10-820.13,820.19, 820.20-820.22, 820.30-820.32, 820.8, 812.00-812.03, 812.09,m 812.10-812.13, 812.19, 812.20-812.21, 812.30-812.31, 812.40-812.44, 812.49, 812.50-812.54, 812.59, 813.00-813.08, 813.10, 813.11-813.18, 813.20, 813.21-813.23, 813.30-813.33, 813.40-813.45, 813.50-813.54, 813.80-813.83, 813.90, 813.91-813.93, 823.00-823.02, 823.10-823.12, 823.20-823.22, 823.30-823.32, 823.40-823.42, 823.80-823.82, 823.90-823.92
Pelvic fractures	808.0-808.3, 808.41-808.43, 808.49, 808.51-808.53, 808.59, 808.8-808.9
Fractures of skull base	801.00-801.06, 801.09, 801.10-801.16, 801.19, 801.20-801.26, 801.29, 801.30-801.36, 801.39, 801.40-801.46, 801.49, 801.50-801.56, 801.59, 801.60-801.66, 801.69, 801.70-801.76, 801.79, 801.80-801.86, 801.89, 801.90-801.96, 801.99
Fractures of face bones	802.0-802.1, 802.20-802.29, 802.30-802.39, 802.4-802.9
Fractures of cervical vertebrae without spinal cord injury	805.00-805.08, 805.10-805.18
Clavicle fracture	810.00-810.03, 810.10-810.13
Stroke and intracranial hemorrhage	997.02, 430, 431, 432.0, 432.1, 432.9

**Table 2:** ICD-9-CM diagnosis codes for hospital complications

Cardiac and MI	997.1, 410.00-410.02, 410.10-410.12, 410.20-410.22, 410.30-410.32, 410.40-410.42, 410.50-410.52, 410.60-410.62, 410.70-410.72, 410.80-410.82, 410.90-410.92, 427.5
Respiratory and pneumonia	997.3x, 480.x, 481, 482.0-482.2, 482.3x, 482.4x, 482.8x, 482.9, 483.x, 484.x, 485, 486, 507.0, 512.1, 518.4, 518.5, 518.81, 518.82
Renal	997.5, 584.x, 593.81
Urinary tract infection	599.0, 996.64
Sepsis and bloodstream infection	038.xx, 415.12, 785.52, 995.91, 995.92, 996.61, 996.62, 998.0, 999.31, 999.39
Surgical site infection	998.31-998.32, 998.51, 998.59
Bleeding	998.11, 998,12, 285.1

SAS 9.4 software (SAS Institute, Cary, NC) was used for data analysis and all statistics. Categorical variables were compared with Chi-square test and multivariable logistic regression analysis with adjustment for patient age, gender, race, and major comorbidities. Because such numeric parameters as LOS and cost were not normally distributed and highly skewed to the right, we compared them with the non-parametric Wilcoxon rank sum test. Two-sided P < 0.05 was considered significant.

## Results

**Fig. 1:**
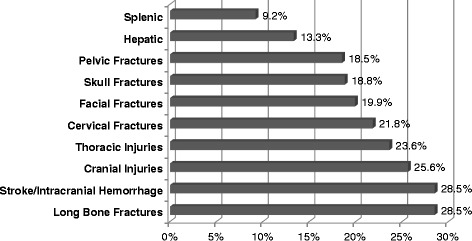
Associated Injuries

1,686,867 patients were estimated having sustained MVC during the time period queried; 1,168 patients with BCI were identified. No patients with carotid dissection underwent an open repair, 4.24 % were treated with carotid stenting, and 95.76 % of patients had no operative intervention. Patients who had sustained a blunt MVC were divided into age groups to evaluate the frequency of BCI after MVC. Age groups were defined as: 18–24 (27.8 %), 47–60 (22.3 %), 35–46 (20.6 %), 25–34 (19.1 %), >61 (10.2 %). From this evaluation it was found that the highest frequency of BCI was found in the age group of 18–24. Associated injuries included long bone fractures (28.5 %), stroke and intracranial hemorrhage (28.5 %), cranial injuries (25.6 %), thoracic injuries (23.6 %), cervical fractures (21.8 %), facial fractures (19.9 %), skull fractures (18.8 %), pelvic fractures (18.5 %), hepatic injuries (13.3 %), and splenic injuries (9.2 %) (Fig. 1). Complications associated with BCI included respiratory (44.2 %), bleeding (16.1 %), urinary tract infections (UTI) (8.9 %), and sepsis (4.9 %). Overall mortality following BCI was 14.1 %. There was no significant difference in mortality between those with and without intervention (18.5 % vs. 13.9 %; *P* = 0.36). Stroke and intracranial hemorrhage was independently associated with a 2.7 time greater risk of mortality. Mean length of stay for patients with BCI undergoing stenting compared to no intervention were similar (13.1 days vs. 15.9 days) but had a greater mean cost ($83,030 vs. $63,200) although the difference in cost was not statistically significant (*p* = 0.307).

## Discussion

Blunt carotid injury remains a rare injury associated with major traumatic events. It can, however, lead to severe consequences with a significant associated rate of stroke and intracranial hemorrhage. This analysis demonstrated that when stroke and intracranial hemorrhage occurred it was associated with a 2.7 times greater risk of mortality. This association has been previously noted and prompted the development of screening criteria noted as the Denver or Memphis criterion [5]. This analysis also illustrates that BCI was more frequent in younger patients, was associated with long bone fractures, thoracic injuries, and pelvic fractures suggesting that the mechanism is more important than the anatomic location of injury. As well, this analysis suggests that carotid stenting was not associated with improved mortality and had increased cost.

Appropriate early identification and treatment is important to help reduce the risk of stroke. Patients meeting criteria are recommended to undergo further evaluation with computed tomography angiography [5, 6]. Early diagnosis and treatment blunt carotid arterial injury has been shown to reduce overall complication rates associated with those injuries [6, 7]. BCI usually starts with an initial tear of the intima. When subendothelial collagen is exposed, it acts as a thrombogenic agent starting the cascade of platelet aggregation and resultant thrombus formation. Although most patients have no overt neurological deficits on diagnosis of the injury, it is well known that there exists a latent period ranging from hours to days during which neurological deficits may manifest in the initially asymptomatic patient [8]. The Memphis screening protocol was adopted to screen for BCI on the presentation of physical injuries associated with carotid artery injury such as basilar skull fractures, cervical spine fractures, Horner’s syndrome, soft tissue injuries of the neck, and neurological symptoms not otherwise explained in brain imaging [9]. Furthermore the mechanism of injury should also raise suspicion for possible BCI especially if rapid deceleration, hyperextension, and/or severe flexion along with rotation of the neck may be involved [2]. This analysis demonstrated what previous authors have described with cervical fractures, facial fractures, and skull fractures being common. Our analysis illustrates that long bone fractures and thoracic injuries occur at even greater rate in this patient population and should be considered as mechanisms associated with BCI. In the event of a diagnosed BCI, it is graded based on the Denver grading scale. While there is currently no standardized treatment algorithm, it is generally accepted that interventional treatment utilizing carotid artery stenting is not initiated until the dissection has progressed to a pseudoaneurysm or in the event of rapid progression of disease and ensuing hemodynamic instability increasing risk for arterial occlusion or transection [10]. Most recently it is advocated that only grade II and grade III injuries be considered candidates for endovascular stenting. Routine stenting is believed to add risk for stroke without any added benefit. Endovascular carotid stenting has been suggested to trap thrombus and reduce further enlargement of the pseudoaneurysm and possibly also the rupture risk [11]. Patients are therefore recommended to undergo heparinization or antiplatelet therapy in case of contraindications to systemic heparinization [6]. Intimal flaps noted on the trauma protocol computed tomography scan are readily and easily identified today’s generation of high quality, multirow detection computed tomography scanners. Once the diagnosis of BCI is made, the optimal treatment is often dependent upon the concomitant injuries. Overall consensus has been that antithrombotic therapy, either with heparinization or antiplatelet agents, has improved outcomes. Randomized controlled trials by Fabian et al. have shown certain benefit of anticoagulation over no treatment of identified BCI with regards to neurologic outcome [7].

Optimal treatment for blunt carotid injury remains unclear. Antithrombotic therapy either with systemic heparinization or with antiplatelet agents has been associated with improved neurologic outcomes [7, 8, 12]. As our study illustrates, BCI is associated with numerous other injuries which can increase the risk of therapeutic anticoagulation. Multiple studies have documented the morbidity associated with full anticoagulation in this patient population with significant rates of complications including intracranial hemorrhage. More common, however, is gastrointestinal and retroperitoneal bleeding as well as solid organ injury causing hemorrhage. Those significantly morbid complications associated with systemic anticoagulation have led to the study of antiplatelet therapy for those whom anticoagulation is deemed contraindicated has been shown to be essentially equivalent [11, 13, 14].

Previous authors have reported that patients with combinations of head, facial, and cervical spine injuries with or without extremity fractures proved to be at significantly increased risk for BCI [15]. This analysis demonstrated that there was a very high incidence of long bone fractures (over 25 %) as well as thoracic injuries (approaching 25 %) suggesting that the majority of patients noted to have BCI were involved in MVC with significant mechanism to produce multisystem injuries. Pelvic fractures were also seen at a rate nearing 20 %. While most pelvic fractures would be noted by the initial pelvic roentgenogram, our study would suggest that further evaluation of extremity complaints should be considered in anyone noted to have evidence of BCI. The rates of long bone fractures and thoracic injuries were higher than cervical fractures, facial fractures, and skull fractures. These observations are notable for the clinicians evaluating trauma patients and may represent the force of the injury.

Our study also illustrates the cost of treating a traumatically injured patient with BCI. Carotid intervention for BCI trended towards in increased hospital utilization. It is notable, however, that patients who sustained injuries with associated BCI have long hospital stays of 13–16 days which speaks to the significance of concomitant injuries in this cohort. In addition, our study showed a high rate of UTI in patients who suffered BCI. This, again, describes the significance of concomitant injuries of these patients that have sustained motor vehicle collision and should be yet another reminder to clinicians to remove urinary catheters early. One would expect that in absence of other major injuries, BCI would be associated with a relatively shorter hospital stay as the majority of injuries are asymptomatic and current recommendations are follow-up imaging studies at one week and three months [16].

### Limitations of the study

The use of an administrative data was originally intended for billing purposes and carries the innate limitation of billing data. As well, the temporal relationship cannot be determined from these data and these are associated findings. For example, stroke and intracranial hemorrhage were associated with BCI, but the order in which the events occurred cannot be determined from these data, only that they were both present during that admission. There is the potential for selection bias based on limited coding schemes. Additionally, due to the large number of hospitals reporting the data and the variability of the individual coders entering the data, there is a potential for coding errors. Of note, given the possible biases, it is often found that coding of injuries and diagnoses such as infectious complications may be reported or under-coded. Refined clinical data is not possible from administrative data; however this study describes rates of complications and outcomes utilizing thousands of patients to evaluate a rare clinical condition.

## Conclusions

Blunt carotid injury remains a rare injury associated with a major traumatic event. Outcomes can be improved by early detection and institution of appropriate treatment which is overwhelmingly medical management with antithrombotic therapy. Establishment of the injury profile which has been described from this analysis should assist the clinician to evaluate or screen for BCI. This study found that intervention trended toward increased costs and no improvement in outcomes was found. In addition, the incidence of long bone, pelvic fractures, and thoracic injuries were significantly associated with BCI and suggest clinicians should consider an evaluation for BCI in these high risk patients.

## Abbreviations

BCI, blunt carotid arterial injury; CAS, carotid artery stent; ICD-9-CM, international classification of diseases, ninth revision, clinical modification; MVC, motor vehicle collision; NIS, nationwide inpatient sample; UTI, urinary tract infections
